# Cardioprotective effects of shock wave therapy: A cardiac magnetic resonance imaging study on acute ischemia-reperfusion injury

**DOI:** 10.3389/fcvm.2023.1134389

**Published:** 2023-04-27

**Authors:** Lorena Petrusca, Pierre Croisille, Lionel Augeul, Michel Ovize, Nathan Mewton, Magalie Viallon

**Affiliations:** ^1^ Univ Lyon, UJM-Saint-Etienne, INSA, CNRS UMR 5520, INSERM U1206, Saint-Etienne, France; ^2^ Department of Radiology, Centre Hospitalier Universitaire de Saint- Etienne, Université Jean-Monnet, Saint-Etienne, France; ^3^ INSERM UMR 1060, CARMEN Laboratory, Université Lyon 1, Faculté de Medecine, Rockfeller, Lyon, France; ^4^ Heart Failure Department, Clinical Investigation Center, Inserm 1407, HCL—Lyon, France

**Keywords:** shock wave therapy, myocardial ischemia/reperfusion injury, acute myocardial infarction, experimental studies, cardioprotection, mechano-transduction, MRI

## Abstract

**Introduction:**

Cardioprotection strategies remain a new frontier in treating acute myocardial infarction (AMI), aiming at further protect the myocardium from the ischemia-reperfusion damage. Therefore, we aimed at investigating the mechano-transduction effects induced by shock waves (SW) therapy at time of the ischemia reperfusion as a non-invasive cardioprotective innovative approach to trigger healing molecular mechanisms.

**Methods:**

We evaluated the SW therapy effects in an open-chest pig ischemia-reperfusion (IR) model, with quantitative cardiac Magnetic Resonance (MR) imaging performed along the experiments at multiple time points (baseline (B), during ischemia (I), at early reperfusion (ER) (∼15 min), and late reperfusion (LR) (3 h)). AMI was obtained by a left anterior artery temporary occlusion (50 min) in 18 pigs (32 ± 1.9 kg) randomized into SW therapy and control groups. In the SW therapy group, treatment was started at the end of the ischemia period and extended during early reperfusion (600 + 1,200 shots @0.09 J/mm2, f = 5 Hz). The MR protocol included at all time points LV global function assessment, regional strain quantification, native T1 and T2 parametric mapping. Then, after contrast injection (gadolinium), we obtained late gadolinium imaging and extra-cellular volume (ECV) mapping. Before animal sacrifice, Evans blue dye was administrated after re-occlusion for area-at-risk sizing.

**Results:**

During ischemia, LVEF decreased in both groups (25 ± 4.8% in controls (*p* = 0.031), 31.6 ± 3.2% in SW (*p* = 0.02). After reperfusion, left ventricular ejection fraction (LVEF) remained significantly decreased in controls (39.9 ± 4% at LR vs. 60 ± 5% at baseline (*p* = 0.02). In the SW group, LVEF increased quickly ER (43.7 ± 11.4% vs. 52.4 ± 8.2%), and further improved at LR (49.4 ± 10.1) (ER vs. LR *p* = 0.05), close to baseline reference (LR vs. B *p* = 0.92). Furthermore, there was no significant difference in myocardial relaxation time (i.e. edema) after reperfusion in the intervention group compared to the control group: *Δ*T1 (MI vs. remote) was increased by 23.2±% for SW vs. +25.2% for the controls, while *Δ*T2 (MI vs. remote) increased by +24.9% for SW vs. +21.7% for the control group.

**Discussion:**

In conclusion, we showed in an ischemia-reperfusion open-chest swine model that SW therapy, when applied near the relief of 50′ LAD occlusion, led to a nearly immediate cardioprotective effect translating to a reduction in the acute ischemia-reperfusion lesion size and to a significant LV function improvement. These new and promising results related to the multi-targeted effects of SW therapy in IR injury need to be confirmed by further in-vivo studies in close chest models with longitudinal follow-up.

## 1. Introduction

After acute myocardial infarction (AMI), prognosis is mainly determined by infarct size (1). Hence interventions to reduce final infarct size have a major clinical interest in improving the prognosis of patients referred for AMI. The current management of myocardial infarction relies on timely myocardial reperfusion by primary percutaneous coronary intervention (PCI). However, experimental and clinical studies have shown that reperfusion, which causes an abrupt supply of oxygen, increases the necrosis of the heart muscle inducing therefore deleterious effects on the ischemic myocardium. Paradoxically, the intervention that saves patients' lives is also responsible for a significant part of heart muscle necrosis. It creates additional injury (so-called reperfusion injury) (RI) (1). Several mechanisms have been identified to mediate myocardial RI. Briefly, those include the oxidative stress and free radicals accumulation within the first minutes, the intracellular calcium overload, the rapid pH changes, and an unclear role of the inflammation response that accompanies an AMI (2, 3).

This post-reperfusion damage increases the final infarct size by up to 40% (4) limiting functional recovery, favoring adverse left ventricular (LV) remodeling, and increasing the risk of heart failure and death.

Despite many propositions of cardioprotective therapies tested in preclinical settings, none of these innovative treatments has provided concrete proof of true clinical effectiveness (3, 5). Most of them have relied on using a single target approach directed at a specific single molecule or intracellular signaling pathway. Experts in the field of cardioprotection have hypothesized that a multi-targeted approach directed towards more than one intracellular pathway and/or different cell compartments or mechanisms (myocytes, micro-vascularization, inflammation, etc.) could be a more effective strategy (3, 6, 7).

One potentially promising cardioprotective approach in AMI is represented by mechanotransduction (MT). Indeed, it has the potential to simultaneously target several intracellular pathways and damage induced by reperfusion (myocardial edema, dysfunction of microcirculation, inflammation).

MT represents a biological pathway to which many cell types are sensitive and can adapt their behavior to external mechanical-acoustic stimulation (8). Recent studies have indicated the importance of MT for various cell types, including fibroblasts, macrophages, and immune system cells involved in inflammation. The biological mechanisms of action based on MT can be activated non-invasively by using shock waves (SW) therapy, clinically proven as a safe and effective approach, initially used for lithotripsy, fracture healing, or treatment for calcifying tendinitis of the shoulder or chronic plantar fasciitis (9–12). MT also plays a strong role in positively influencing cell functions and homeostasis, enhancing tissue self-healing capabilities (8).

Since the most prominent effect of the mechanical stimulus of SW therapy on tissues was to induce regenerative effects (13, 14), research on this emerging therapeutic strategy has been focused on chronic myocardial ischemia. Experimental studies have used mainly coronary total occlusion models (15–20) with SW therapy delivered within the first week and the impact evaluated at a chronic stage after 1 month. In parallel, small clinical trials have tested SW therapy with promising results in advanced stages of coronary artery disease with symptomatic relief in patients with refractory angina (21–23), without side effects, while promoting neo-vascularization in border regions and inhibiting apoptosis (24–26).

Nevertheless, and while the mechanism of SW efficacy appears to rely on multiple actions, a strategy now specifically sought to benefit from a maximized effect on ischemia reperfusion (IR), there are currently no existing preclinical or clinical results on the immediate effects of SW therapy at the onset of the acute ischemia reperfusion injury (IRI), either during acute ischemia or within the first hours of reperfusion on the acutely injured myocardium.

Therefore, our main objective was to investigate the acute effects of SW therapy using quantitative MRI at the acute phase of MI and evaluate therapy benefits in function or changes in the tissue leading to final myocardial damage in an experimental myocardial reperfusion-injury model.

## 2. Material and methods

### 2.1. Animal model and experimental procedures

The study protocol was approved by the State Committee on Animal Affairs of our Institution and conformed to Directive 2010/63/EU of the European Parliament. The swine model was designed to allow us to test SW therapies at the earliest minutes after reperfusion with adjustable lesion-size depending on the coronary occlusion site.

A total of 19 animals, with 32 ± 1.9 kg body weight, were included in this study. The animals were randomized into 2 equivalent groups (*n* = 9, 4 male, 5 female per group), with intervention (SW group) and without (Control group) (more details in supplementary materials). In addition, considering that safety data already reported the absence of procedural complications or detectable relevant side effects after SW therapy, only one animal was used as a “sham” to optimize the experimental conditions, with no induced ischemia, but SW therapy applied with the same parameters as in the therapy group.

*Animals' preparation, anesthesia, and monitoring.* The animals were initially premedicated with intramuscular administration of ketamine (15 mg/kg), xylazine (2 mg/kg) and acepromazine (0.3 mg/kg). Following placement of continuous monitoring for heart rate, electrocardiogram (ECG), respiration, oxygen saturation and end tidal CO2 (EtCO_2_), propofol (5 ml at 1%) and fentanyl (10 µg/kg) were given intravenously. Each pig was initially intubated with a 7.5 to 8 mm cuffed endotracheal tube and mechanically ventilated with a time cycled, limited pressure ventilator (peak inspiratory pressure (PIP) 18 cmH_2_O, positive end-expiratory pressure (PEEP) 5 cmH_2_O, rate 30 breaths/min, inspiratory to expiratory ratio (I/E) 1:1) delivering a gas mixture containing sevoflurane (3% concentration), to maintain adequate anesthesia, and a fractional inspiratory oxygen concentration (FiO_2_) of 30% in the air mixture. Central arterial and venous catheters were placed for continuous low perfusion (5–10 ml/h) of a mixture of ketamine, xylazine, and fentadon, given to avoid any painful perception of the animal. Throughout the whole procedure and including cardiac MR imaging, we monitored and registered continuously the body temperature, heart rate and arterial pressure, oxygen saturation, and EtCO2.

In case of LV fibrillation, direct current external defibrillation was applied immediately outside the MR room. At the end of the protocol, the animals were sacrificed without awakening by injection of T61 euthanizing agent (0.3 ml/kg).

#### 2.1.1. Acute ischemia-reperfusion model

Experimental myocardial acute ischemia was performed using an open chest swine model via coronary artery temporary ligation (27). The chest was opened by surgical median sternotomy, allowing direct access to the heart. A vessel loop was carefully tied around the left anterior descending (LAD) artery. The occlusion was performed by tying the lace around the LAD artery for 50 min while avoiding any damage, at the most proximal possible position on the LAD to induce a large area at risk (AAR). Visual verification by local myocardial cyanosis and immediate akinesia, together with ECG changes, proved an efficient infarction after ligation. After 50 min, the lace was released to allow complete reperfusion. After the de-occlusion, the reperfusion of the LAD was considered satisfactory if the artery was visualized as successfully recanalized. Care was taken to verify that no introduced bias could have arisen from anatomical difference in the coronary bed occluded explaining the differences between groups.

#### 2.1.2. Shock-wave (SW) protocol

Cardiac SW therapy was performed with the SW DUOLITH device (Storz Medical, Switzerland). We set the administrated SW energy in the ischemic myocardium to 0.09 mJ/mm^2^ delivered at a frequency of 5 Hz over a total of 1,800 shots (15, 17). SW was applied to the ischemic myocardium for 2 min (equivalent to 600 shots) at the end of the ischemic period and was pursued during 4 min after reperfusion, (equivalent to 1,200 shots). The sham experiment respected an identical SW protocol but did not apply the IR procedure. The device gel pad was positioned directly in contact with the myocardium, using ultrasound gel for acoustic coupling. The device was continuously moved along the infarcted area and surrounding tissue, following and compensating the 3D cardiac contractions while delivering SW. We paid particular attention to ensuring permanent contact with the myocardial surface to avoid attenuation of the delivered energy.

#### 2.1.3. Postmortem tissue analysis

Before the animal sacrifice, we performed a LAD re-occlusion at the exact same location, and 100 ml of Evans Blue was injected into the jugular vein to identify the AAR, so that the size of the non-bluesified area could be later quantified. The heart was explanted, sliced and high-resolution digital pictures of resulting *ex vivo* heart slices were recorded. Care was taken so that the central slice corresponded to the imaged MR slice in short axis (SAX). All slices were individually weighted. The AAR was calculated after manual segmentation of the unstained areas in each macroscopic digital pictures so that the AAR could be provided in grams (g) and % of the LV mass. Acute IR lesion size was calculated from cardiac magnetic resonance (CMR) and late Gadolinium imaging data (28), as detailed below.

### 2.2. CMR data acquisition

**Figure 1:**
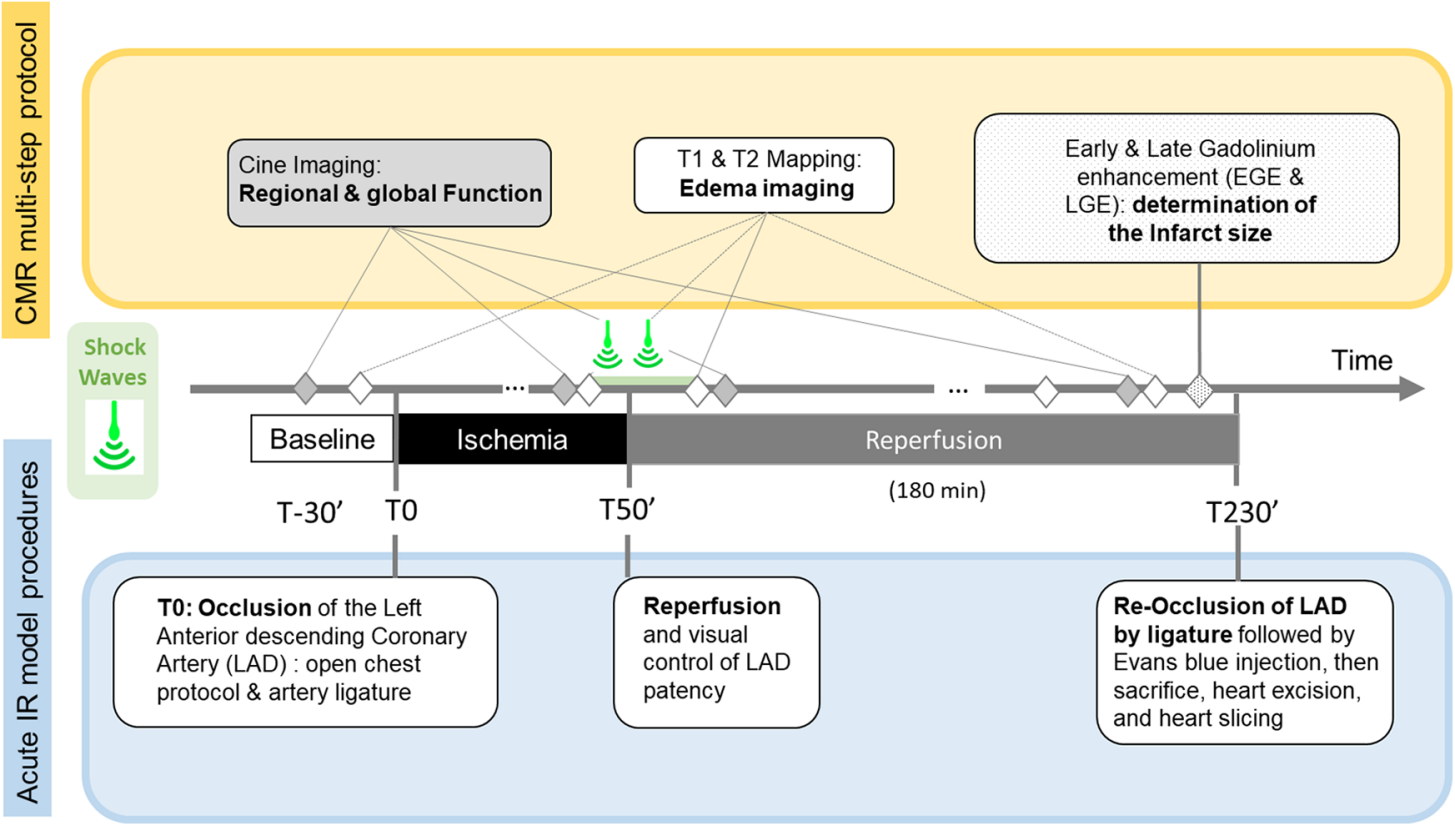
Timeline of the Cardiac Magnetic Resonance (CMR) multi-step protocol (top), Shock-Wave (SW) therapy time points, and acute Ischemia/Reperfusion (IR) open chest swine model stages.

We performed all CMR examinations on a 1.5 T system (MAGNETOM Avanto, Siemens Healthineers, Erlangen, Germany). A 6-element surface coil was operated in an integrated fashion with the Spine Matrix coil (2 rings of 6 elements each) composing a 12-elements design. Figure 1 summarizes how the MR protocol, consisting of repeated quantitative MRI (QMRI) and function protocol, performed at main time-points, precisely synchronized with the key steps of the experimental procedure: Baseline (B), Ischemia (I), Early Reperfusion (ER) after SW therapy (∼ after 15 min of reperfusion), and late reperfusion (LR), i.e., after 3 h of reperfusion) and finally, after Gadolinium administration(post-Gd) (10 min delay, 0.2 mmol.kg^−1^, Dotarem, Guerbet, France).

The function and QMRI protocol included native T1, and T2 mapping in SAX (performed approximately in the middle of the infarcted area), CINE acquisitions in SAX and long axis (LAX, 2 and 4 chamber-view, LVOT), all acquired during individual and repeated breath-holds. For the function protocol, 10 to 15 SAX stack acquisitions covering without gap the whole LV from basis to apex were acquired in a single breath-hold using the compressed sensing (CS) technique (29, 30). Details on MR sequences parameters can be found in Table 1 in supplementary materials.

**Table 1:** Main MR derived parameters obtained in control and intervention (SW therapy) groups, at the main stages of the protocol.

		Control group				SW Therapy group			
		Baseline	Ischemia	Early Reperfusion	Late Reperfusion	Baseline	Ischemia	Early Reperfusion	Late Reperfusion
T1 Native Remote (ms)		1,024.5 ± 18.2	1,039.5 ± 12.6	1,046.3 ± 16.8	1,036.7 ± 19.8	1,041.8 ± 35.9	1,056.7 ± 36.6	1,074.1 ± 19.7	1,059.6 ± 25.6
T1 Native Lesion (ms)		1,046.7 ± 40.6	1,141.6 ± 26.5	1,292.5 ± 88	1,277.4 ± 117.9	1,036.7 ± 21.4	1,102.3 ± 28.4	1,331 ± 31.2	1,293.9 ± 57.2
T1 Native Blood (ms)		1,799.3 ± 80.9	1,864.7 ± 75	1,818.5 ± 80.6	1,838.8 ± 102.6	1,795.5 ± 66.8	1,856.5 ± 91.8	1,858.5 ± 85.6	1,830 ± 90.1
T2 Remote (ms)		48.9 ± 2.5	50.4 ± 1.2	49.8 ± 2.1	49.6 ± 2	49.1 ± 2.3	49.4 ± 1.9	49.4 ± 2.4	50.1 ± 2.7
T2 Lesion (ms)		49.1 ± 3.2	53.3 ± 1.6	59.4 ± 4.2	60.4 ± 5.9	49 ± 0.8	49.5 ± 1.5	62.3 ± 5.2	61.3 ± 4.8
T2 Blood (ms)		215.7 ± 26.0	239.8 ± 15.9	222 ± 13.2	227 ± 21.5	205.9 ± 25.7	209.1 ± 17.9	220.3 ± 14.3	226.1 ± 15.8
*Δ*T2 (ms)		0.2	2.9	9.6	10.8	−0.1	0.2	12.9	11.2
*Δ*T1 (ms)		22.3	102.1	246.3	240.8	−5.1	45.6	256.9	243.3
*Δ*T2 (%)		0.4	5.8	19.2	21.8	−0.2	0.3	26.1	22.3
*Δ*T1 (%)		2.2	9.8	23.5	23.2	−0.5	4.3	23.9	22.1
%SE_T2_		–	8.6	20.9	23.1	–	1.1	27.1	25.2
%SE_T1_		–	9.1	23.5	22.0	–	6.3	28.4	24.8
LVEF (%)		60 ± 5	25.5 ± 4.8	36.4 ± 7	39.7 ± 4	52.4 ± 8.2	31.6 ± 3.2	43.7 ± 11.4	49.4 ± 10.1
LV Mass (g)		90.3 ± 10.1	85.7 ± 3.7	96.7 ± 13.4	96.5 ± 11.5	85.6 ± 13.4	89.5 ± 12.3	98.5 ± 14.8	106.1 ± 10.4
EDV (ml)		58.1 ± 26.6	65.0 ± 7.8	66.6 ± 25.3	67.4 ± 19.2	47.1 ± 10.8	60.2 ± 10.2	49.2 ± 6.3	47.2 ± 9.9
ESV (ml)		23.9 ± 13.9	47.5 ± 2.4	43.0 ± 17.6	41.2 ± 13.2	22.4 ± 6.8	41.3 ± 8.2	27.8 ± 7.4	24.3 ± 8.5
*E*_*cc*_ (%)	lesion	−14.4 ± 4.7	−5.6 ± 0.4	−0.5 ± 1.6	1.7 ± 4.6	−9.2 ± 4.2	−0.2 ± 6.3	0.7 ± 5.0	1.8 ± 3.8
	remote	−11.1 ± 2.5	−6.3 ± 5.0	−10.5 ± 5.2	−11.3 ± 3.3	−14.7 ± 7.6	−10.8 ± 6.2	−14.9 ± 6.8	−11.1 ± 6.8
	border	−10.71 ± 4.0	−7.74 ± 1.1	−7.28 ± 5.6	−6.76 ± 4.6	−11.00 ± 5.2	−7.18 ± 2.9	−10.35 ± 5.9	−8.75 ± 4.6

data are mean ± SD; *Δ*T1 and *Δ*T2 are lesions vs. remote regions contrast in ms and %; % SE_T1_ and % SE_T2_ are % signal enhancement over time within the lesion; LVEF: left ventricular ejection fraction; LV: left ventricle; EDV: end-diastolic volume: ESV: end-systolic volume; *E*_*cc*_: Lagrangian circumferential strain.

### 2.3. Image analysis

#### 2.3.1. Quantification of IR lesion size and no-reflow region

The 3D stack of Late Gadolinium Enhancement (LGE) images were processed using CMRSegTools software (v 1.5.1, CREATIS, Lyon, France) (31) to get the total volume of the hyperintense myocardium on LGE, corresponding to the acute IR lesion, expressed in grams (g) and as a percentage of total LV mass. The % of no-reflow (NR) was calculated from the same images (defined as hypointense areas located within hyperintense areas), and also expressed as a percent of the total MI-mass (%). These measures were performed bearing in mind that no-reflow occurs in 40%–57% of AMI patients with a worsened prognosis (32, 33), and that therapies applied at the time of reperfusion may help to limit a microvascular obstruction (34).

#### 2.3.2. T1, T2 and extracellular volume quantification

On the parametric SAX T1 and T2 maps, the lesion area, defined as infarcted tissue (including no reflow region, if any), and the remote area, considered as normal tissue, were delineated on each map at all time-points of the protocol. The mean T1 and T2 values were extracted from these Regions of Interest (ROIs) and from ROIs positioned in the cavity (blood) and were stored for further analysis. The time evolution of the MR indexes for all animals in each of the compartments of interest: lesion, blood, and remote myocardium were derived. From T1 values measured on native and post-gadolinium T1 maps, and using an hematocrit obtained from blood drawn immediately before Gadolinium injection, we further calculated the extracellular volume (ECV) estimate in remote and border regions (35).

#### 2.3.3. *Ex vivo* myocardial water content

Representative samples of the IR lesion and of the remote regions were weighed before (wet weight), and after drying to a constant weight (24 h at 100°C) in a desiccating oven. The percent myocardial water content was calculated as: (wet weight—dry weight) × 100/wet weight (36).

#### 2.3.4. Global and regional function quantification

Post-processing of CINE MR images was performed using Circle Cardiovascular Imaging software (CVI42 version 5.13, Calgary, Canada). All data sets were analyzed by a consensus of 2 experienced experts.

The left ventricular end-diastolic and end-systolic inner endocardial and outer epicardial contour segmentations were obtained on all CINE stacks of images. Papillary muscles were systematically included in the cavity. LV volume-based parameters included End Systolic Volume (ESV (ml), End Diastole Volume (EDV, (ml)), myocardial mass (LV mass, g), and Left Ventricular Ejection Fraction (LVEF, %) calculated for all time points.

**Figure 2:**
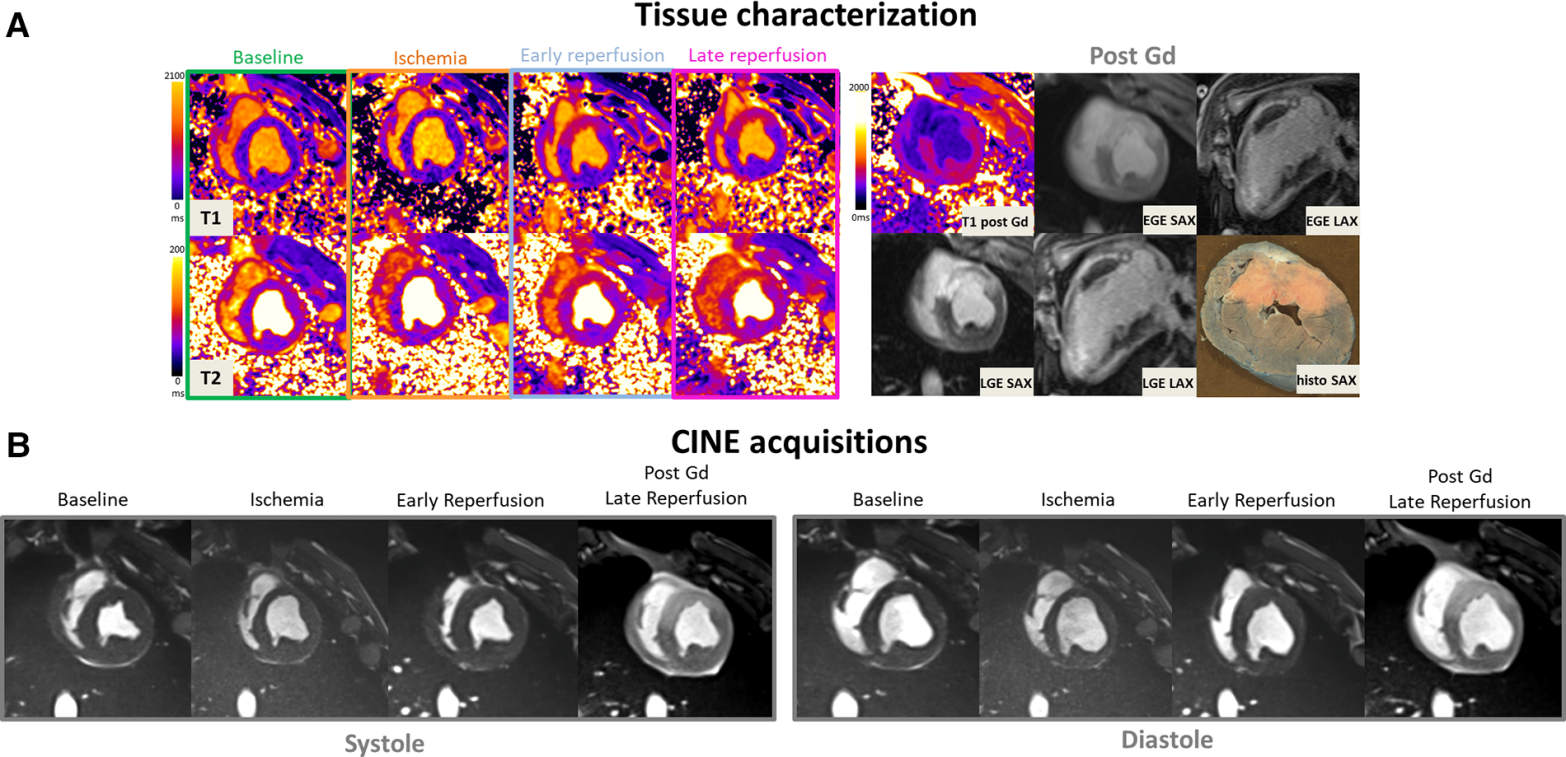
(**A**: top left) Representative short-axis T1 and T2 maps obtained at baseline, during ischemia, early, and late time-points of the reperfusion in a swine open chest model from the treated SW group. (**B**) CINE bSSFP images are displayed in short axis, in diastole and systole at different time-points. (**A**: top-right) Early and late Gadolinium enhanced images (EGE and LGE) matching to the corresponding T1 maps, together with *ex vivo* macroscopic corresponding mid-ventricular slice after Evans blue dye are also displayed (top-right). Gd hyper-enhanced sectors in post-contrast bSSFP images can be easily identified, visually matching the EGE and LGE hyperintense areas. These sectors correspond to akinetic segments during ischemia but also where edema immediately and clearly appeared after reperfusion on both T1 and T2 maps.

Strain analysis was conducted using the feature-tracking approach with CVI42 package and applied to CINE acquisitions to calculate the Lagrangian circumferential strain (Ecc). Endocardial and epicardial segmentation was performed at end-diastole and then automatically propagated throughout the whole cardiac cycle. The end-systolic phase was determined using the LV volume curve to obtain end-systolic circumferential strain values. To minimize partial volume effects, we divided the myocardium into 12 segments, then selected three representative regions: core lesion, border, and remote regions. The regions were chosen based on post-contrast CINE acquisitions obtained at the end of reperfusion. Indeed, Gd hyper-enhanced sectors in post-contrast bSSFP images can be easily located (see Figure 2). Remote regions were typically chosen on the opposite regions, with preserved contractile function, and without Gd enhancement. Border regions were sectors located immediately outside of the hyper-enhanced regions while ensuring there was no partial volume with damaged hyperenhanced tissue. This classification of sectors in CINE series was obtained at baseline, ischemia, and early reperfusion and further conducted using anatomical landmarks in SAX and LAX to ensure the best possible matching (see supplementary material for details).

### 2.4. Statistical analysis

Data were screened for normality using the Shapiro-Wilk test and reported as mean ± standard deviation (SD) or median and 95% confidence interval [CI 95%]. Unpaired t-tests with Welch's correction when required were used to compare IR lesion, AAR size, as well as ECV differences among the two groups. A two-way repeated measures mixed-effects model approach with a compound symmetry covariance matrix and REstricted Maximum Likelihood (REML) fitting was run to determine the effect of SW therapy (treatment) and time (during the ischemia reperfusion procedure and follow-up) on LVEF, EDV, ESV, and mass. A third factor was used further to determine the effect of location (region effect) when analyzing T1, T2, and strain measures. A Geisser-Greenhouse correction was applied to account for sphericity assumption violation. Pairwise post-hoc comparisons a Tukey test to correct for multiple comparisons when applicable. For all analyses, significance was accepted at *P* < 0.05. The statistical analyses were conducted using Prism 9 (La Jolla, CA, USA) and Stata 17 (College Station, TX) statistical analysis packages.

## 3. Results

We included a total of 19 animals in this study. Four animals died before the end of the protocol (1 during ischemia, three during reperfusion). One animal was used as a sham to verify the SW safety applied directly on the myocardial tissue with IR. Seven out of 9 animals were successfully treated after IR using SW, and 7 out of 9 also survived in the control group. Hemodynamic profiles along procedure were similar in both groups and a similar occurrence of ventricular arrhythmias (including VT and VF) was reported in both groups.

**Figure 3:**
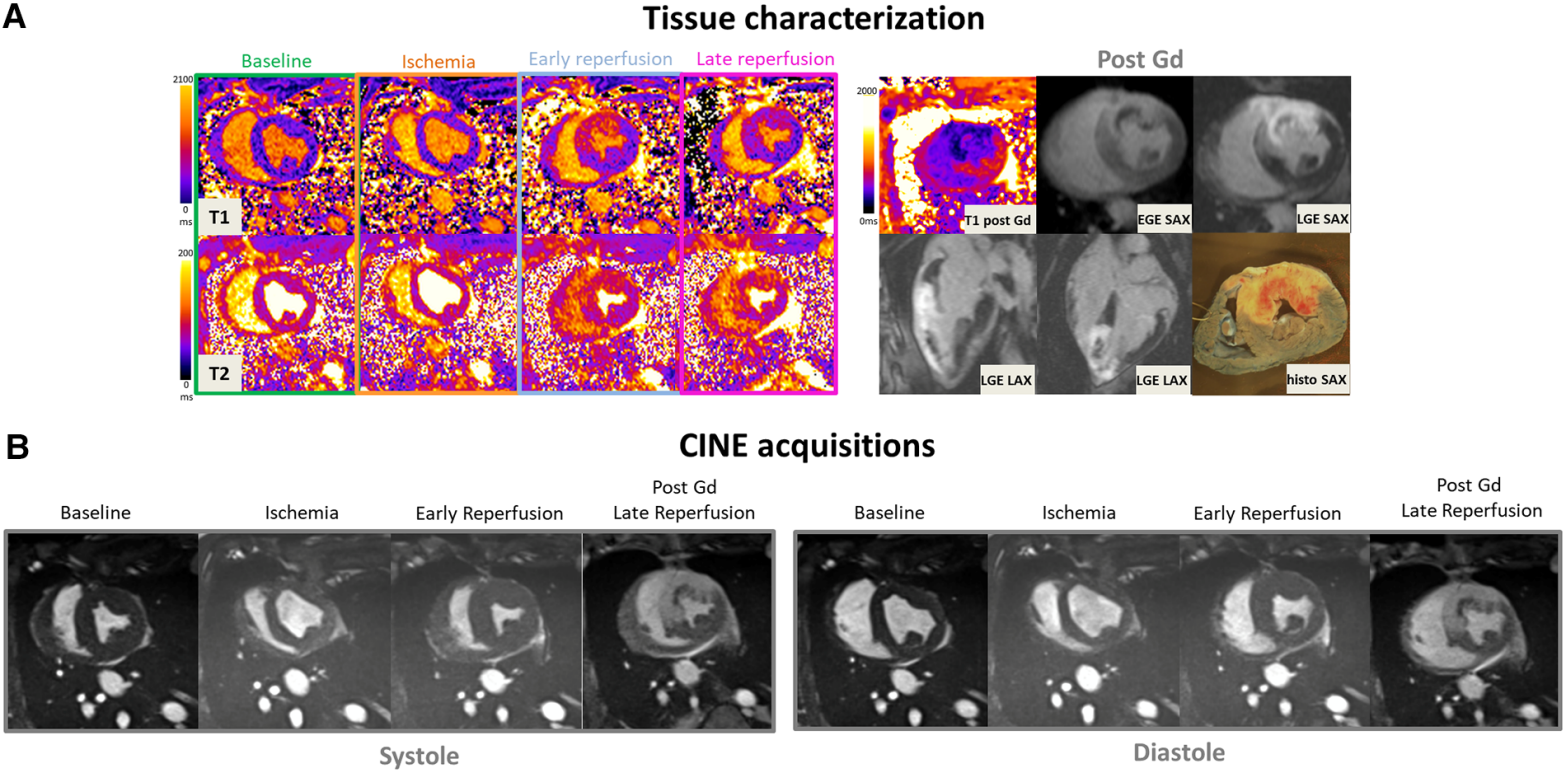
(**A**: top left) Representative short-axis T1 and T2 maps obtained at baseline, during ischemia, early, and late time-points of the reperfusion in a swine open chest model from the control group. (**B**) CINE bSSFP images are displayed in short axis, in diastole and systole at different time-points. (**A**: top-right) Early and late Gadolinium enhanced images (EGE and LGE) matching to the corresponding T1 maps, together with *ex vivo* macroscopic corresponding mid-ventricular slice after Evans blue dye are also displayed (top-right). Gd hyper-enhanced sectors in post-contrast bSSFP images can be easily identified, visually matching the EGE and LGE hyperintense areas. These sectors correspond to akinetic segments during ischemia but also where edema immediately and clearly appeared after reperfusion on both T1 and T2 maps.

Figure 2 provides a representative example in one SW-treated animal at the different time points of the protocol showing physiopathology changes in T1 and T2 maps, CINE images in diastole and systole, EGE and LGE maps and corresponding *ex vivo* macroscopic appearance after Evans Blue staining. Similarly, Figure 3 illustrates an example of control animal.

### 3.1. Ischemia-reperfusion (IR) lesion size, AAR and *ex vivo* imaging

There was not significant difference between controls and the SW group in the ischemia extent with an AAR of respectively 29.9[24.8; 34.9] % and 22.8[10.9; 34.8] % of the total LV mass (mean difference 7[−51.8; 19.2], *p* = 0.22).

While the acute IR lesion size was 27.3[21.7; 32.9] % of the LV in controls, it was reduced to 20.8[16.3; 25.5] % of the LV in the SW therapy group (mean difference 6.44[0.11; 12.77], *p* = 0.047) (Supplementary Material Figure S2). When calculated as % of the AAR, IR lesion size was 91.5[80.8; 102.2] in controls, and 78.9[55.9; 101.9] in the SW therapy group (mean difference −12.6[−36.3;11.08], *p* = 0.25].

No-reflow regions were observed in only a small number of animals and were very limited in size: in the SW group: 2.14 ± 1.8% (*n* = 3), and in controls: 3.58% (*n* = 1).

### 3.2. Global function

**Figure 4:**
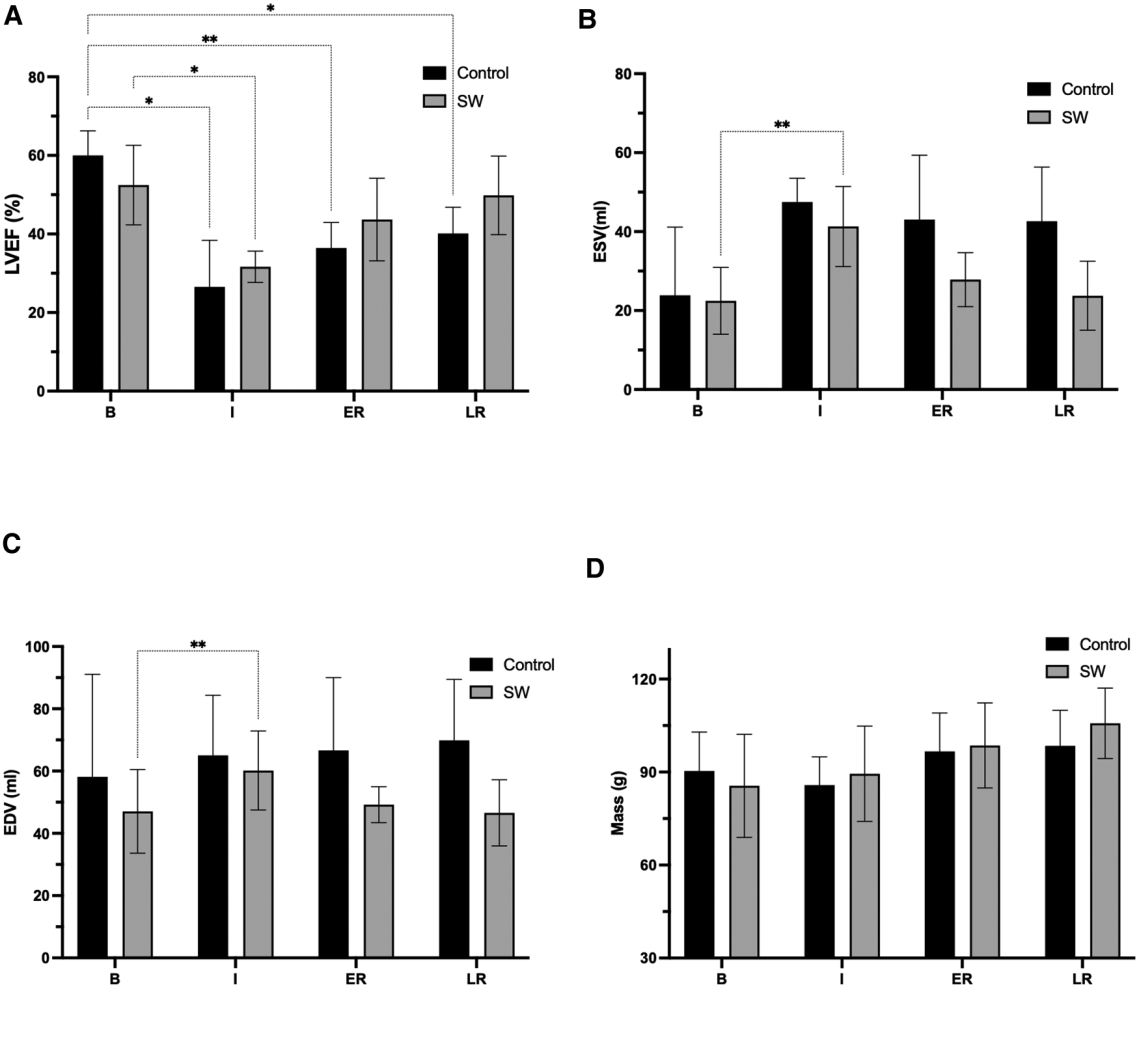
Global function parameters (LVEF (**A**) ESV (**B**), EDV (**C**), and LV mass (**D**)) changes at the four stages of the experimental ischemia-reperfusion protocol: Baseline (**B**), Ischemia (**I**), Early (ER) and Late Reperfusion (LR)), in controls (black) and SW therapy group (grey). (*: *p* < 0.05, **: *p* < 0.01). LVEF: left ventricle ejection fraction, ESV: end-systolic volume, EDV: end-diastolic volume, LV: left ventricle. All post-hoc comparisons are provided in Supplementary Material Section, Table S2.

All derived function parameters are listed in Table 1. During ischemia, LVEF decreased in both groups, with a mean difference from their baseline of −33.4[−59.5;−7.4]% (*p* = 0.031) in controls and −21[−36.6;−4.9]% (*p* = 0.020) in SW group (no significant difference between control and SW group (*p* = 0.57)). After reperfusion, while LVEF in controls remained significantly decreased compared to baseline both at early reperfusion (−23.5[−36.8;−10.3]% (*p* = 0.007)) and late reperfusion time points (−19.8[−33.7;−5.9]% (*p* = 0.015), global LV function in SW-therapy group improved significantly at early reperfusion with a mean difference compared to baseline values of −8.7[−22.9;5.5]% (*p* = 0.19). In the SW group, LVEF continued to improve at late reperfusion and returned nearly to baseline values (mean difference of −2.6[−20.6;15.4]% (*p* = 0.86)). See Figure 4 and Supplementary Material Table S2 (Supplemental Materials) for details.

There were no significant acute LV end-diastolic changes, according to the lack of significance of overall changes in end-diastolic volumes over time (*p* = 0.28) or across groups (0.07). In the control group, there was a trend toward increasing EDV values over time, reaching their maximum at late reperfusion +11.7 [−16.6;40.1] ml. In the SW group, there was a temporary increase in EDV during ischemia (+13.1 [7.9;18.3] ml), returning to nearly baseline values after early reperfusion (+2.1 [−20.9;25.2] ml and at the late reperfusion time point (−0.5 [−37.7;36.7] ml). In controls and compared to baseline values, ESV increased during ischemia +23.6 [−4.6;51.9] ml (*p* = 0.07] and remained nearly unchanged after reperfusion (at late time point:+18.7 [−4.9;41.9] ml (*p* = 0.09)). In SW group, ESV increased temporary during ischemia (+18.8 [7.2;30.4] ml (*p* = 0.01), and returned to +5.3 [−9.3;20.1] ml (*p* = 0.49) and +1.3 [−20.3;22.9] ml (*p* = 0.98).

LV end-diastolic mass increased with time (*p* = 0.008) with a trend toward higher values after reperfusion, but without difference among groups.

### 3.3. Regional strain

**Figure 5:**
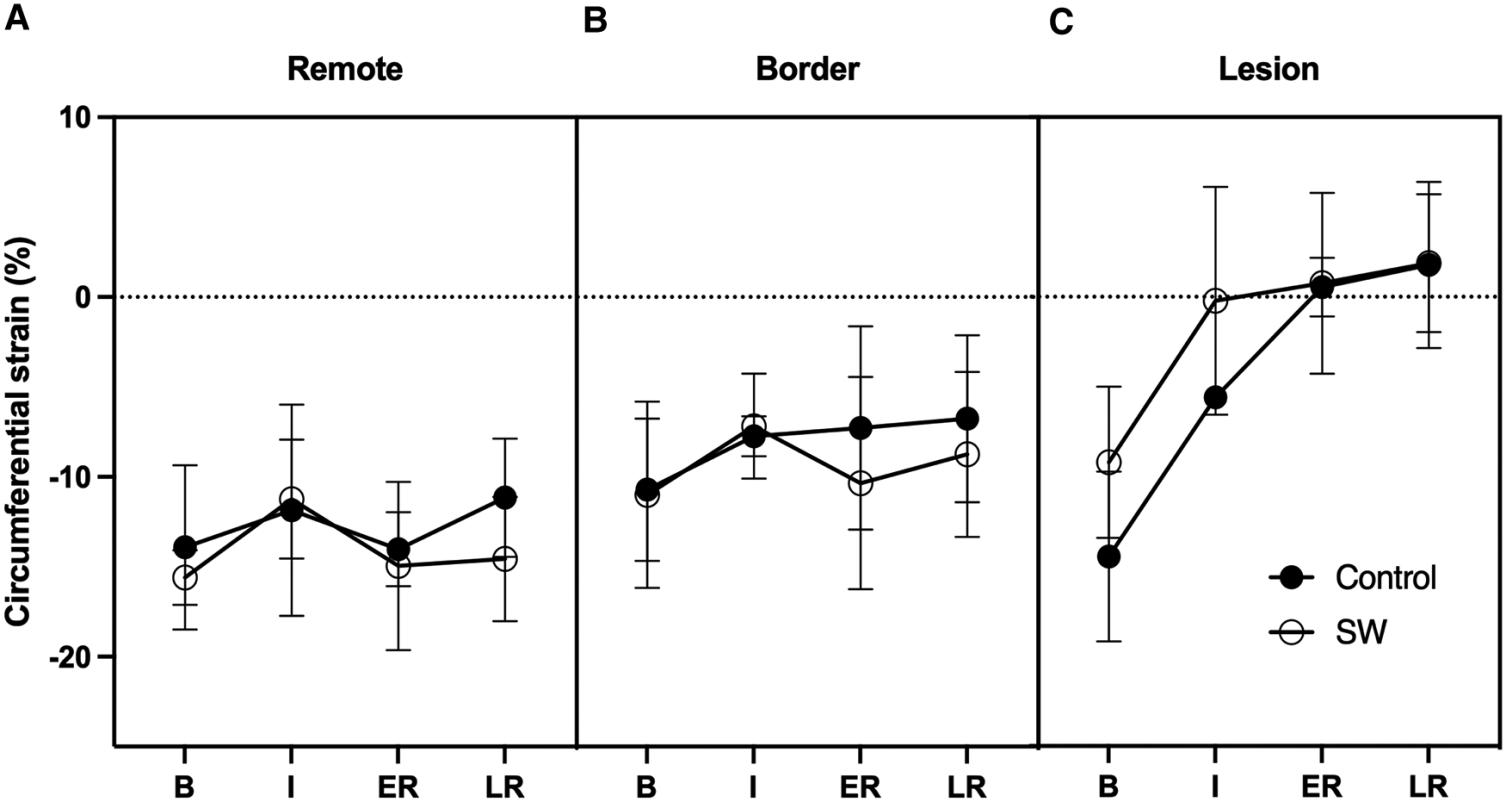
Circumferential regional Strains time-course at Baseline (**B**), Ischemia (**I**), Early (ER) and Late Reperfusion (LR) in Control Group (black dots) and SW Therapy group (white dots). Data are provided in remote (**A**), border (**B**) and lesion (**C**) regions of the central SAX slice.

In the core lesion, regional strain (Ecc) was severely impacted during IR (*p* < 0.001), with most changes occurring at ischemia (mean difference from baseline +10.1 [5.3,14.7] %), with a further trend toward stretching over reperfusion (+3.6% at late reperfusion (*p* = 0.23)). There was no significant treatment effect (*p* = 0.22) and no interaction between these 2 factors (*p* = 0.29) (Figure 5).

In remote and border regions, there was no significant effect of time (*p* = 0.13) or treatment (*p* = 0.5), but a trend to slightly improved Ecc strain values during reperfusion between SW and controls (−3% in ER in border, −3.4% in LR in remote regions; *p* = NS).

### 3.4. T1, T2, and ECV quantitative measures

We found a significant effect of time on T1 and T2 (both *p* < 0.0001) as well as differences among regions (remote vs. lesion) (*p* < 0.0001), but no overall effect of treatment (control vs. SW) (T1: *p* = 0.44; T2: *p* = 0.91)) (also see Table 1).

Compared to baseline values, during ischemia the increase of T1 in lesions (mean difference[95 CI%]) was +95[50;140]ms (*p* = 0.0014) in the control group and +66[19;112]ms (*p* = 0.01) in the SW group. After reperfusion, T1 measures reached their maximum amplitude changes at the early reperfusion time point, with +246[75;416]ms (*p* < 0.009) in controls and +294[220;369]ms (*p* < 0.001) in the SW group. In late reperfusion, T1 increase was +231[53;409]ms (*p* = 0.016) in controls and +257[182;332]ms (*p* < 0.001) in SW group.

During ischemia and compared to baseline values, the increase of T2 in lesions was +4[0.4;8.1]ms (*p* = 0.034) in the control group and +0.55[−1.7;2.8]ms (*p* = 0.77) in the SW group. After reperfusion, early T2 changes were +10.3[2.5;18.1]ms (*p* = 0.016) in controls and +13.3[5.6;21.0]ms (*p* = 0.004) in the SW group. In late reperfusion, T2 increase was +11.4[−1.5;24.2]ms (*p* = 0.07) in controls and +12.3[4.9;19.8]ms (*p* = 0.005) in SW group.

The moderate increase of T1 and T2 during ischemia in the lesion area was followed by a marked increase after reperfusion in both groups. The rise in T1 and T2 indexes that quantify the presence of edema in the tissue demonstrate similar changes after reperfusion and similar differences between the remote and lesion area in both the control and treated group: *Δ*T1 (MI vs. remote) = 259.9 ms for SW (+24,6%) vs. 258.2 ms (+25.2%) for the controls, while *Δ*T2 (MI vs. remote) = 12.3 ms for SW (+24,9%) vs. 10.8 ms (+21.7%) for the control group.

However, the difference between the normal tissue and the ischemic tissue was more important during the phase of ischemia in the control group: *Δ*T1 (MI vs. remote) = 46.6 ms (+4.44%) for SW vs. 102.1 ms (+9.82%) for the controls, while *Δ*T2 (MI vs. remote) = 0.5 ms (+0.96%) for SW vs. 2.9 ms (+5.78%) for the controls. (R->I) = 26.9%).

ECV in remote regions was 24.7[23.1; 26.3]% in controls and 26.1[23.7; 28.5]% in SW group (*p* = 0.25). In border regions there was only a trend toward higher values in the SW group, with respectively 25.6[17.9; 33.3]% and 30.4[19.4; 41.4]% (*p* = 0.40).

The *ex vivo* myocardial water content was signiﬁcantly higher in the lesion than the remote myocardium both in the SW treated group (86.6 ± 3.2% vs. 81.1 ± 1.1%; *p* = 0.001) and in the control group (84.7 ± 3.0 vs. 79.5 ± 3.1%; *p* = 0.001). The myocardial water content in the lesion and in the remote myocardium were slightly higher in the SW group than in the controls (respectively *p* = 0.56 and *p* = 0.47).

## 4. Discussion

We explored the effects of non-invasive cardiac SW therapy in an acute ischemia reperfusion swine model (open-chest, 50 min LAD occlusion, 3 h of reperfusion) using a longitudinal quantitative CMR follow-up at four specific time points.

Our main findings are the following: 1) after SW therapy and reperfusion, there was a significant and early improvement of global systolic LV function (increase in LVEF and ESV, return to baseline of EDV at 15' of reperfusion) that further improved after 3 h of reperfusion. 2) strain imaging showed that regional contractile function remained severely altered in the core lesion. In the border and remote segments, there was only a trend to higher contractility in SW-treated animals; 3) SW therapy did not significantly modulate the amount of myocardial edema within 3 h of reperfusion.

### 4.1. Global LV function changes

An essential finding of our study is the early recovery of LVEF following SW therapy (SW was applied at the end of ischemia for 2 min + 4 min immediate after artery opening) in the treated group to nearly baseline values (*p* = NS), observed as early as ∼15 min after reperfusion (ER imaging time point). LVEF was, as expected, significantly decreased during ischemia in both groups. Of note is the further improvement observed 3 h after reperfusion in the treated group, while in the control group, the mean value remained significantly below baseline (Figure 4). LVEF improvement resulted from a normalization of ESV (= recovery of contractile function) and the absence of acute LV dilatation after reperfusion in the treated group.

To our knowledge, this is the first demonstration of an acute and immediate positive effect of SW therapy at the onset of an ischemia-reperfusion injury. While most efforts have been concentrated on unraveling the chronic benefits of SW therapy, this novel finding was possible only because we chose to explore what was happening during ischemia and after reperfusion at different time points. These findings also take advantage of the capabilities of CMR to quantify and monitor LV function changes at the different endpoints together with tissue characterization. CMR is indeed recognized as the gold standard method in several measurements, including the quantification of LV volumes, EF, and LV mass in clinical routine but also in clinical research since taking advantage of the highest reproducibility and enabling dramatic sample size reductions compared to echocardiography for detection of similar changes with comparable power (37). Furthermore, recent recommendations have advocated CMR endpoints in myocardial infarction experimental and clinical trials to improve the transferability and translation of experimental results to the clinic (28).

Most mechano-transduction (MT) research has focused on chronic myocardial ischemia because an obvious clinical target was patients with refractory angina with end-stage myocardial ischemia. Pre-clinical animal studies assessed the chronic benefits of SW therapy, usually evaluated 4 weeks after SW therapy application. Nishida used a chronic ischemia model (ameroïd constriction over 4 weeks), followed by SW therapy and evaluation performed 4 to 8 weeks. They found a complete recovery to baseline LVEF values (15). Holfeld et al. used a coronary ligation model. After 4 weeks of recovery, SW therapy was applied after re-thoracotomy. While LVEF decreased to 43% following coronary ligation (baseline LVEF 67%), LVEF returned also to baseline values 4 weeks after treatment while there was no improvement in control animals (20).

### 4.2. Regional strains

Circumferential strains alone failed to demonstrate the same pattern as LVEF. Still, it should be noted that circumferential strains returned nearly to baseline level in border regions, similar to what happened in remote regions. When integrated into the whole parts of the myocardium, these changes likely compensated for the loss of function in the irreversibly damaged tissue. In the core lesion, strains in the SW therapy group matched those of controls, likely due to the severity of the ischemic damage obtained after 50 min of LAD occlusion leading to nearly transmural lesions (see Figure 2). Nevertheless, while in the control group, there was a worsening of the regional strains at 3 h of reperfusion in the remote and the ischemic lesion, there was a trend to strains remaining stable in the lesion while improving in the remote region. We used end-systolic strain instead of peak strain to describe the impact of the acute ischemia-reperfusion procedure locally since peak values are known to overestimate regional function due to the passive recoil leading to post-systolic shortening even in myocardium with transmural lesions when LV pressure is failing during isovolumic relaxation (38). Circumferential strain was selected as recognized as the most robust and reproducible strain components with CMR imaging (39, 40) and segmental analysis allows to distinguish areas of sub-endocardial from transmural infarction (41, 42).

### 4.3. Myocardial edema

Water content measured by T1 and T2 relaxometry locally in ischemic and remote regions increased globally and to a greater extent in damaged tissue of both groups. T2 has been validated against pathology for myocardial water content in experiment MI models (28, 43). At the same time, T1 is also sensitive to other molecules and is advocated a further marker of infarct core (44), which did not occur when comparing SW therapy and controls. Note that LV end-diastolic mass increased over time and reached its maximum at LR (+7.6 ml or +8% in controls, + 19 ml or +23.6% in SW compared to baseline) is likely related to the global increase in the whole heart's water content, which can be explained by the vasodilator effect across targeted tissues and the modulation of the inflammatory response induced by SW therapy. This is also supported by the increase of the extracellular volume (ECV), that was +5.7% in remote regions in the SW group, and +18.6% in the border regions (*p* = 0.40).

Indeed, several mechanisms of action have been identified as signaling pathways known to be activated in SW therapy and may explain SW cardioprotective effects detailed recently by Graber et al. (45): 1) the enhancement of blood flow related to the generation of nitric oxide (NO) as a potent vasodilator (46, 47); 2) the activation of the innate immune receptor Tool-Like receptor 3 (TLR3) that triggers an early pro-inflammatory response (48), but modulate late anti-inflammatory response (49). Other signaling pathways are also combined to explain the observed benefits of SW therapy in sub-acute or chronic models: 3) upregulation of anti-apoptotic proteins (overexpression of BCL-2), and downregulation of pro-apoptotic proteins, while preventing activation of components of the mitochondrial-dependent intrinsic apoptotic pathway (50, 51); 4) induction of the release of angiogenic growth factors (including VEGF, fibroblast growth factor (FGF) and others (52–54); 5) endothelial and mesenchymal progenitor cells chemoattraction via stromal-derived factor (SDF-1) release (55, 56) combined with activation purinergic receptors enhancing stem cell proliferation (57).

One hypothesis that would be worth to be tested in future studies could be to repeat the delivery of the SW in additional sessions along the reperfusion period in order to evaluate whether if could help to maintain or further improve the mechanical capacities of the myocardium at higher levels. This dynamic slow-down is also in agreement with the arterial pressure (PA) variation during the reperfusion period (PA^C^ = 82 and PA^T ^= 82.6 at the end of reperfusion, while PA^C^ = 82.7 and PA^T ^= 87.9 during ischemia) and can most probably be related to the model itself.

Following a sustained episode of lethal IR process (50 min in our case), one major determinant of MI size is the AAR of infarction, which is easily identified at the end of the acute protocol. Here, macroscopic evaluations of AAR do not indicate apparent differences between the two groups. When evaluating the 3D MR LGE data: the total IR-reperfusion lesion, including the NR volume, was calculated to be 27.3% of the LV mass in the control group vs. 20.8% in the treated group. While an enhanced area in LGE images is the clinical standard for assessing myocardial lesion size in chronic infarct (58), at this hyperacute stage, the reduced hyperintense lesion observed in the treated group must also be interpreted with respect to the post-reperfusion inflammatory context (28, 59, 60). When assessing the infarct size shortly after ischemia-reperfusion, note that the volume of distribution of the Gadolinium tracer relates to the irreversibly damaged tissue increased in size by cell swelling and extracellular edema that may result in lesion size larger than the AAR in some cases.

The study design included 2 groups (SW therapy and control groups), but no sham group. While SW therapy only requires the gentle contact of the transductor onto the myocardium, and the delivered SW energy was set within the low energy range (0.09 mJ/mm^2^), we nevertheless tested the appropriateness of our specific setup in a sham animal that underwent the whole SW group protocol except the LAD occlusion itself. This subject was used to verify our ability to properly apply SW in open chest swine heart, which is anatomically rather differently positioned, while confirming the innocuity of SW application, the absence of arrythmia or any symptoms prior going on with the acute myocardial infarct model. For the sake of transparency, we decided to mention it as a “sham” and that we did not get any observable side effects in that animal.

We did not observe any side effects, macroscopic, imaging-related tissue damage, or suspect quantitative parameters changes that could be related to SW-related injury. In addition, we did not observe in any of the animals any finding that could be related to additional tissue damage in the SW group. This confirms the recent seminal work of Pölzl et al. (61) that clarified the therapeutic range for SW therapy, and justify SWT doses currently used in ongoing clinical trials on myocardial ischemia. They demonstrated, both *in vitro* and experimentally in mice, that SW therapy does not induce cellular damage beneath energy levels of 0.27 mJ/mm2 total flux density, while endothelial cell proliferation, angiogenic gene expression are induced dose-dependently in a therapeutic range between 0.07 and 0.15 mJ/mm2 energy flux density. Also, in the existing literature using SW therapy with the same SW parameters in pigs (acute MI without reperfusion and SW application within the first week), all authors reported the absence of procedural complications or detectable relevant side effects.

## 5. Limitations of the study

The total number of animals included in the study (*n* = 9/group, *n* = 7 analyzed) is relatively small. All the animals followed an identical procedure and monitoring within a strictly defined protocol. Due to ischemia-induced VF events that typically occur during I and ER steps in open chest swine models, external shocks were delivered in 6 animals of each group with no side-effects directly on the myocardium, and no exclusion was performed. Moreover, halogenated gases including sevoflurane have been shown to have cardioprotective properties (62, 63). Since both groups received the same anesthesia protocol, we believe it cannot be considered as a bias that could negate the validity of the presented results.

The energy flux density, the frequency, and the number of impulses applied have been derived from other experimental studies (15, 64) and were invariable for all experiments. Therefore, even if a benefit is demonstrated here, the present data did not evaluate the SW dose-depended effects of the therapy, due to the multiple parameters that can be varied. Besides the SW parameters, the number of therapy sessions and/or different time-points for SW delivery could be explored according to the size and severity of the ischemic zones. It could play a key role in tailoring personalized treatments. However, a dose-dependent evaluation of clinical parameters was beyond this article's scope. Moreover, for long-term cardioprotective efficacy evaluation of the novel therapy, extended periods of reperfusion (>24–72 h) are required, and therefore a chronic animal model is needed. Also, a chronic model avoids the initial edematous reaction occurring during the first week, and allows optimal measurement of the final infarct in % of AAR, set as the reference standard for cardioprotection studies.

Our study did not yield histological markers that could have helped to refine our understanding of the observed response in treated vs. untreated individuals. Indeed, we designed the protocol to get the same MRI endpoints as those used in patients in AMI clinical trials. This strategy has been advocated in recent position papers (3, 28) to improve the transferability of experimental results of new developments to humans. MRI endpoints are, for most of them, surrogate measures that have already been validated against pathology, including T1 (65, 66) or T2 mapping against pathology for myocardial water content in experimental models (43, 67). Beyond the functional changes, we were particularly interested in exploring the role of water changes and edema occurring after reperfusion and the impact of SW therapy. MRI is the only approach that can quantify non-invasively these changes in-vivo at multiple time points, with the animals serving as their own control. T1 and T2 relaxation times are increased in the presence of edema, with T1 being also sensitive at later times to degradation products in hemorrhage, such as deoxyHb. ECV is derived from pre- and post-gadolinium T1 measures. All these approaches are considered complementary and recent recommendations are to use both techniques, in particular in experimental settings or clinical trials (see (28)). For quality control of the T1, T2, and ECV measures, we performed an *ex vivo* quantification of the water content in AAR and remote myocardium using the conventional wet/dry weighting approach of tissue samples (36). As expected, these measures corroborate the imaging findings but exclude the ability to obtain histological markers.

One of our hypotheses was also that the emission of acoustic waves (shockwaves) that carry energy and propagate through tissues could relieve the pressure exerted by edema on the capillaries, generating interstitial and extracellular water drainage, helping local reperfusion and reducing reperfusion injury. Unfortunately, no-reflow regions were observed in only a few animals, and further studies are needed to investigate this aspect further.

Finally, in a clinical perspective, the applicability, and the scenarios of use of SW therapy in the scope of acute myocardial ischemia will depend on whether SW therapy need to be applied directly on the epicardium, or if transthoracic transducers are a valid option. Transthoracic SW therapy has however to face known limitations: small acoustic window, application restricted to anterior myocardium, potential lung injuries (68), but, if successful, may open this technique to the largest number of patients.

In conclusion, we showed in an ischemia-reperfusion open-chest swine model that SW therapy, when applied near the relief of 50' LAD occlusion, led to a nearly immediate cardioprotective effect translating to a reduction the acute lesion size and to a significant LV function improvement. The immediate mechanical benefit occurred 15' after reperfusion and was observed in all treated animals compared to the control group. This study suggests that SW may be a promising, effective, and safe cardioprotective approach that should be further considered in acute ischemia settings in addition to existing chronic ischemia clinical scenarios. Further work is needed to have a comprehensive understanding on how mechanical stimulation act on the at-risk myocardium. While CE and/or FDA extracorporeal SWT devices are already available and designed for clinical applications, they suffer from intrinsic limitations, including a reduced acoustic window for optimal application in all myocardial regions. The benefit of an epicardial approach during surgical procedures may be the optimal solution as proposed in ongoing clinical CAST-HF trial (69) while limiting the range of clinical scenarios of applicability.

If our present findings are confirmed in future studies, the use of the cardiac SW therapy may be used as an initial treatment in patients with AMI or tested in repeated in sessions in further clinical trial studies. Moreover, its design enables it to be used complementary with other treatment types. Its clinical implementation could be straightforward since the SW device used is already homologated for clinical use if the myocardium and its benefits reported in published studies illustrating the use of SW in cardiac therapy in chronic myocardial ischemia.

## Data Availability

Data generated and analyzed during the study are not publicly available. Requests to access the datasets should be directed to magalie.viallon@creatis.insa-lyon.fr.
