# Supplementary material for: Cardioprotective effects of shock wave therapy: A cardiac magnetic resonance imaging study on acute ischemia-reperfusion injury
Source: Front Cardiovasc Med. 2023 Apr 27;10:1134389. doi: 10.3389/fcvm.2023.1134389 (PMC10172681; doi:10.3389/fcvm.2023.1134389)
Supplement: Supplementary file 1 [file Datasheet1.pdf]

Supplementary Material

Cardioprotective Effects of Shock Wave Therapy: A Cardiac Magnetic Resonance Imaging Study On Acute Ischemia-Reperfusion Injury

Lorena Petrusca[1], Pierre Croisille[1,2], Lionel Augeul[3], Michel Ovize[3,4], Nathan Mewton[3,4], Magalie Viallon[1,2,*]

[1]Univ Lyon, UJM-Saint-Etienne, INSA, CNRS UMR 5520, INSERM U1206, CREATIS, F-42023, Saint-Etienne, France;

[2]Department of Radiology, Centre Hospitalier Universitaire de Saint- Etienne, Université Jean-Monnet, France ;

[3]INSERM UMR 1060, CARMEN Laboratory, Université Lyon 1, Faculté de Medecine, Rockfeller Lyon ;

[4] Heart Failure Department, Clinical Investigation Center, Inserm 1407, HCL - Lyon, France.

*** Correspondence:** Corresponding Author: magalie.viallon@creatis.insa-lyon.fr

1. Animal sample size

The individual swine was considered as the experimental unit within the study. The sample size calculation was based on the number of pigs needed to evaluate and compare the different groups with the statistical power necessary to validate this study, given an expected inter-individual variability similar to humans, and based on the accuracy of MRI measures of the area at risk, edema size, and infarct size observed in human studies. The minimum number to identify changes over time is estimated to be 16, using a conservative risk with α set at 0.05, an expected power (1-□) of 0.95, and a 2-sided assumption for a difference between groups and time points (Stata17). We enforced randomization by alternating control and SW therapy individuals, as well as sex. While no exclusion criteria was applied before the experiment, we excluded retrospectively the animals that died before the end of the protocol (1 during ischemia, 3 during reperfusion). Since statistical tests are based on the accuracy of imaging markers but do not take into account differences in the specific physiological variability of the animals compared to human nor the instability of the model and their prognosis, which remained poorly documented for open chest studies in MRI, we added one more animal per group and one sham.

2. Supplementary Table S1: Main MR Imaging Acquisition Parameters

Parameters	T1-Mapping	T2-Mapping	LGE/EGE	Cine
Sequence Type	MOLLI-5(3)3 pre Gd<br>MOLLI-4(1)3(1)2 Post Gd	T2prep-SSFP	3D-TurFlash	b-SSFP
Readout type	single-shot cartesian	single-shot, cartesian	multi-shot, cartesian	multi-shot, cartesian
Breathing	single breath-hold per slice	single breath-hold per slice	single breath-hold	
FOV	250*250	288*360	220*350	
Slice Thickness (mm)	5	7	4/5	5
Acq. Pixel size (mm^2)	1.3 * 1.3	2.0*1.9	1.0×1.0/ 1.6×1.6	1.0x1.0
Interp. Pixel size (mm^2)	1.3 * 1.3	1.9*1.9		
Voxel size (mm^3)	8.45	26.6	4/12.8	5
Interp. Voxel size (mm^3)	8.45	25.27		
Matrix size	256*168	192*144		
Readout time (msec)	325	239.4		
Repetition time (msec)	Variable TI intervals	4RR	300-400* /240-300<br>563/479	-
Echo time (msec)	1.21	1.06	1.82/1.2	1.39
T2prep (msec)	-	1.06/30/60	-	
Flip Angle(°)	35	35	40°	69
Bandwidth (Hz/Px)	1085	1184	362/1078	930
Averages	1	1	1	
Number of TI/TE	11/ 1	0/3	1/1	
Kspace lines/RR	84	76	51	
Fat suppression	None	None	Fat saturation/ Gradient reversal	None
Grappa factor	2	2	2	2
Partial Fourier	7/8	6/8	6/8	-

3. Supplementary Figure S1: Classification of sectors in CINE SAX series

Example of segments selection (lesion, remote and border) on CINE post-contrast images (left side) and the circumferential strain evolution (right side) during a complete cardiac cycle at the four stages of the experimental ischemia-reperfusion protocol: Baseline, Ischemia, Early and Late Reperfusion

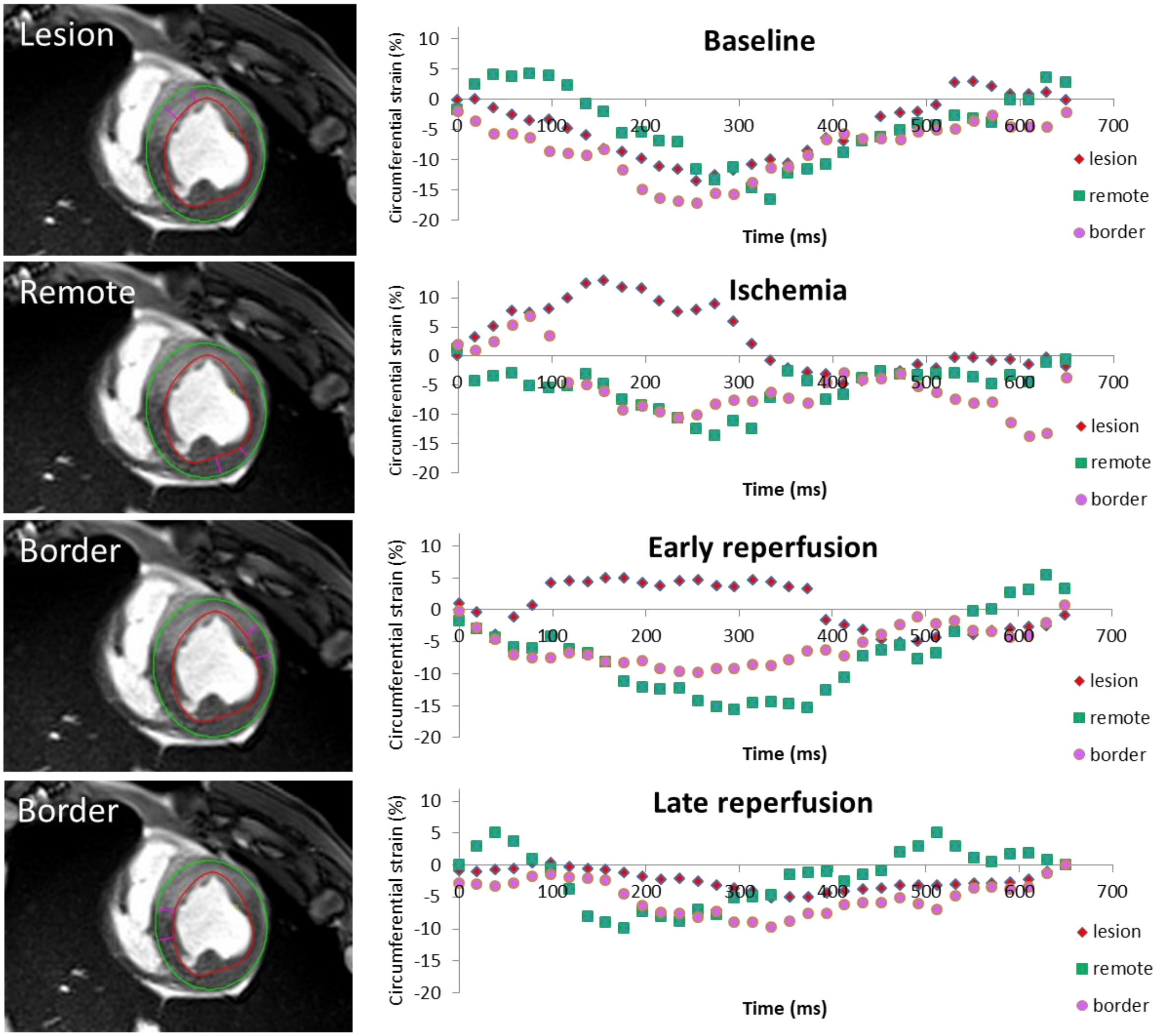

4. Supplementary Figure S2: AAR and IR lesion size

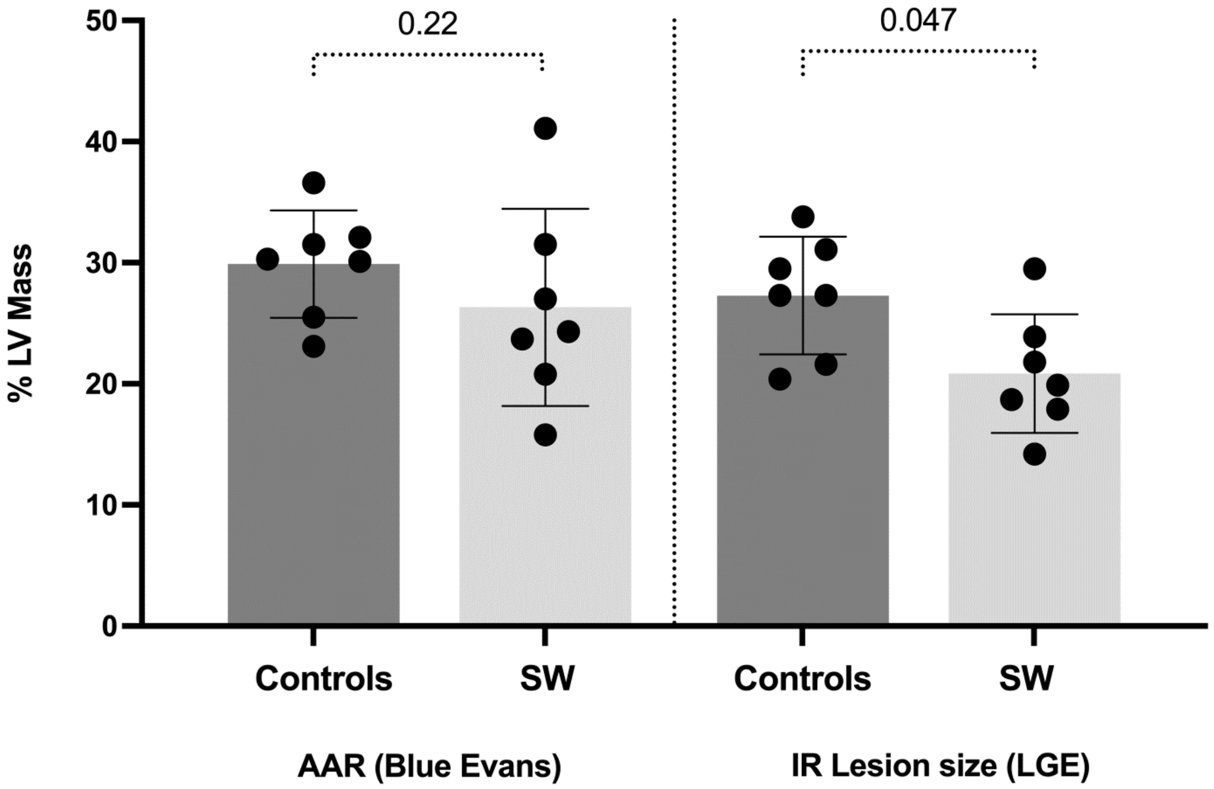

5. Supplementary Table S2: Mixed-effects analysis – multiple post-hoc comparisons for LVEF, ESV, EDV, et LV mass. *(Bold are P values <0.05 and are highlighted differences in Figure 4. I :ischemia ; B : baseline ; ER : early reperfusion; LR: late reperfusion)*

**LVEF**			
Tukey's multiple comparisons	Mean Difference	95% CI of difference	Adjusted P Value
**Control group**			
I vs. B	-33,45	-63,25 to -3,641	**0,0401**
ER vs. B	-23,55	-38,44 to -8,664	**0,0103**
LR vs. B	-19,84	-35,45 to -4,224	**0,0224**
ER vs. I	9,895	-14,33 to 34,12	0,2468
LR vs. I	13,61	-11,87 to 39,09	0,1583
LR vs. ER	3,714	0,4613 to 6,967	**0,0288**
**SW therapy group**			
I vs. B	-20,78	-38,71 to -2,847	**0,0308**
ER vs. B	-8,74	-24,88 to 7,398	0,2642
LR vs. B	-2,623	-23,19 to 17,95	0,9208
ER vs. I	12,04	-4,409 to 28,49	0,1268
LR vs. I	18,16	-4,579 to 40,89	0,0891
LR vs. ER	6,117	-0,02672 to 12,26	0,0508

**ESV**			
Tukey's multiple comparisons	Mean Difference	95% CI of difference	Adjusted P Value
**Control group**			
I vs. B	23,62	-8,778 to 56,02	0,0907
ER vs. B	19,16	-9,731 to 48,06	0,1644
LR vs. B	18,73	-7,451 to 44,92	0,1348
ER vs. I	-4,457	-77,40 to 68,49	0,9691
LR vs. I	-4,886	-52,53 to 42,75	0,8862
LR vs. ER	-0,4286	-7,297 to 6,440	0,9961
**SW therapy group**			
I vs. B	18,84	5,678 to 32,00	**0,0147**
ER vs. B	5,369	-11,34 to 22,07	0,6041
LR vs. B	1,29	-23,36 to 25,94	0,9932
ER vs. I	-13,47	-35,70 to 8,755	0,2051
LR vs. I	-17,55	-51,12 to 16,02	0,2316
LR vs. ER	-4,079	-11,01 to 2,851	0,2496

(Table S2:cont'd)

EDV			
Tukey's multiple comparisons	Mean Difference	95% CI of difference	Adjusted P Value
**Control group**			
I vs. B	6,893	-80,57 to 94,36	0,9398
ER vs. B	8,503	-23,48 to 40,48	0,7175
LR vs. B	11,7	-20,22 to 43,63	0,5167
ER vs. I	1,61	-108,8 to 112,0	0,9995
LR vs. I	4,81	-70,91 to 80,53	0,9657
LR vs. ER	3,2	-7,415 to 13,82	0,7326
**SW therapy group**			
I vs. B	13,12	7,227 to 19,01	**0,0029**
ER vs. B	2,154	-23,95 to 28,26	0,9851
LR vs. B	-0,4933	-42,97 to 41,98	>0,9999
ER vs. I	-10,97	-36,10 to 14,17	0,3984
LR vs. I	-13,61	-54,01 to 26,78	0,4806
LR vs. ER	-2,648	-11,88 to 6,582	0,7266

LV Mass			
Tukey's multiple comparisons	Mean Difference	95% CI of difference	Adjusted P Value
**Control group**			
I vs. B	-4,54	-60,43 to 51,35	0,9788
ER vs. B	6,331	-5,605 to 18,27	0,3223
LR vs. B	8,103	-5,490 to 21,70	0,2427
ER vs. I	10,87	-49,65 to 71,40	0,7232
LR vs. I	12,64	-38,31 to 63,59	0,5233
LR vs. ER	1,771	-3,805 to 7,348	0,8459
**SW therapy group**			
I vs. B	3,9	-10,63 to 18,43	0,8425
ER vs. B	13,03	-3,064 to 29,12	0,1005
LR vs. B	20,16	-12,05 to 52,36	0,1696
ER vs. I	9,126	-6,477 to 24,73	0,2547
LR vs. I	16,26	-24,29 to 56,80	0,4295
LR vs. ER	7,131	-5,394 to 19,66	0,3207
